# Rethinking Hidradenitis Suppurativa Management: Insights into Bacterial Interactions and Treatment Evolution

**DOI:** 10.3390/antibiotics13030268

**Published:** 2024-03-17

**Authors:** Faith D. Huynh, Giovanni Damiani, Christopher G. Bunick

**Affiliations:** 1 Yale School of Medicine, New Haven, CT 06510, USA; faith.huynh@yale.edu; 2 Department of Biomedical, Surgical and Dental Sciences, University of Milan, 20122 Milan, Italy; 3 Italian Centre of Precision Medicine and Chronic Inflammation, 20122 Milan, Italy; 4 Department of Dermatology and Program in Translational Biomedicine, Yale School of Medicine, New Haven, CT 06520, USA

**Keywords:** acne inversa, biofilm formation, anaerobes, antibiotics, bacteria, resistance, biologics, JAK inhibitors, dermatology, inflammatory skin disease

## Abstract

Hidradenitis suppurativa (HS), or acne inversa, is a chronic inflammatory dermatological condition characterized by painful and recurrent nodules and purulent abscesses. HS can have a devastating impact on the quality of life of patients. This condition is commonly localized to the axilla, groin, perineal, and inframammary regions, and can develop fistulas and sinus tracts over time. Its pathogenesis remains elusive and is best characterized at the moment as multi-factorial. Additionally, questions remain about the role of cutaneous dysbiosis as a primary HS trigger or as a secondary perturbation due to HS inflammation. This article features works in relation to HS and its interplay with bacterial microflora. We address current treatment approaches and their impact on HS-related bacteria, as well as areas of therapeutic innovation. In the future, disease-modifying or remittive therapy will likely combine an advanced/targeted anti-inflammatory approach with one that effectively modulates cutaneous and deep tissue dysbiosis.

## 1. Introduction

Hidradenitis suppurativa (HS), also known as acne inversa, is a chronic inflammatory dermatological condition that consists of painful and recurrent nodules and purulent abscesses, mainly affecting terminal hair follicles. This disease commonly affects intertriginous regions such as the axilla, groin, perineal, and inframammary. Over time, fistulas and sinus tracts can develop and exacerbate the disease. Hyperkeratosis, hair follicle inflammation, scarring, and an altered skin microbiota can also be noted in HS patients. Current evidence supports the follicle-centered theory that infundibular hyperplasia can cause occlusion and dilatation of the hair follicle, thus allowing static keratin, cellular debris, and bacteria to collect, leading to inflammation caused by different infiltrates [1,2]. During this process, cysts are also formed. Cyst and hair follicle ruptures release their contents into the dermis, inciting a variety of inflammatory cascades. Macrophages produce proinflammatory mediators such as IL (Interleukin)-1β, TNF (Tumor Necrosis Factor), IL-6, and CXCL8 (C-X-C Motif Chemokine Ligand). These mediators then induce expression of IL-8, CXCL11, CCL2, CCL20, CXCL1, and CXCL6, which attracts neutrophils, T cells, and monocytes into the dermis. This leads to cyst or tendril disintegration and ultimately tunnels [1,2].

Although there is ambiguity about which activation pathways are triggered in HS, there appears to be local complement activation within lesions that ultimately leads to the activation of C5a (complement), an anaphylatoxin that attracts other macrophages and cells to the region [2]. In a 2018 study featuring 54 HS patients and 14 healthy patients, Kanni et al. discovered elevated serum C5a and C5b-9 in those with HS. Based on this study, an overactivation of the complement system may play a role in HS pathogenesis [3].

In addition, antimicrobial peptides (AMPs) may also play a role in HS in relation to the innate immune system. Studies reflect an upregulation of human β-defensin 2 as well as S100A7-9 proteins. Along with their antimicrobial properties, defensins serve as immunomodulators that facilitate the movement and concentration of certain proinflammatory molecules. S100 proteins also have antimicrobial properties against gram-positive and gram-negative bacteria. Although the etiology of these overexpressed AMPs is unclear, there appears to be a correlation between HS skin and the dysregulation of these specific AMPs [4].

The release of bacteria and keratin fragments also results in T and B cell activation, which ultimately causes the inflamed nodules and abscesses seen in HS flare ups. The presence of B cells and plasma cells in particular has been found to increase as HS lesions progress from early to chronic. Although the role of B cells in HS is currently ambiguous, B cells from HS-derived blood expressed elevated levels of CD180 and CCR7. Analysis of HS skin samples also revealed expression of B cell lineage markers MZB1 and MS4A1, which were both undetected in their healthy skin counterpart [5]. In addition, plasma cells are especially abundant in HS lesions and are responsible for the production of immunoglobulins that ultimately form immune complexes [2]. Autoantibodies present appear to correlate with identified Hurley stages; van Straalen et al. provide the following examples: autoantibodies against citrullinated fibronectin and filaggrin in stage 1; ApoE (apolipoprotein E), vimentin, and fibrinogen A in stage 2; and histone 2B (H2B) and tenascin C1 in stage 3 [2].

Epigenetic studies highlighted a dysmethylation background for HS patients, which disrupts cell aging and functionality, inflammatory response, and drug metabolism, consequently altering the cutaneous immune system–microbiome homeostasis [6,7,8,9]. Thus, a more detailed understanding of cutaneous dysbiosis occurrence is of paramount importance to prevent disease progression and innovate a curative intervention instead of merely treatment. Towards this goal, it is critical in HS to develop a validated definition of dysbiosis derived as a true difference from controls and to further elucidate differences between colonization and infection, both of which happen in HS patients.

**Figure 1:**
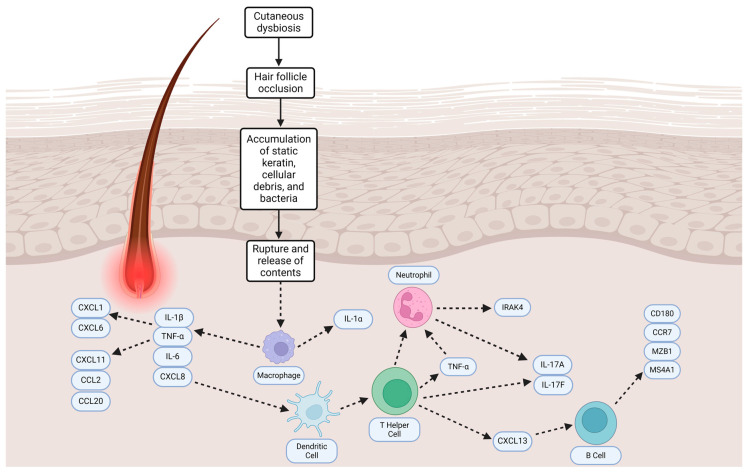
Overview of HS pathogenesis. Created with BioRender.com.

Currently, HS is still an ambiguous and heterogeneous disease as there are varying theories regarding its pathogenesis and relationship with bacteria. It is currently unknown whether the permanent alteration of the skin microbiome is a primary event or a secondary event in response to other factors such as biofilm formation and inflammation. This review discusses the pathogenesis of HS, the efficacy and resistance of antibiotics used in treatment, and the evolved use of and innovation in biologics and advanced systemic small-molecule therapies for HS. Figure 1 depicts HS pathogenesis as supported by current evidence.

## 2. Review

### 2.1. Dominant Bacteria in Skin of HS Patients

The skin microbiota is a crucial component of HS as well as its progression and presentation. The microbiome of patients diagnosed with HS vastly differs from that of healthy, non-HS individuals regarding commensal, opportunistic, and pathogenic bacteria. It is important to define what bacteria are typically found in HS microflora as different antibiotics offer varying spectrums of coverage. HS skin appears to present with a greater abundance of mixed anaerobes and higher diversity in comparison with healthy skin. The relative abundance of anaerobes also appears to positively correlate with HS disease severity in reference to HS Hurley stages [10].

It is generally thought that healthy skin predominantly presents gram-positive aerobic bacteria, such as *Staphylococcus epidermidis* and coagulase-negative *Staphylococci* (CoNS), as well as facultative anaerobic bacteria, such as *Corynebacterium* spp. It has also been known to present with low amounts of strict anaerobes, such as *Cutibacterium*, and gram-negative anaerobic rods, such as *Prevotella*. *Cutibacterium*, formally known as *Propionibacterium acnes*, is a skin commensal known to ferment carbohydrates into propionic acid, which is a compound known for its bactericidal activity against pathogens [11]. By metabolizing free fatty acids from sebum, *C. acnes* is able to decrease surface pH to support commensal bacteria while inhibiting pathogenic bacteria and preventing biofilm formation [12]. Its noted abundance in healthy skin and lack thereof in HS skin may be tied to the pathogenesis of HS. Recent studies indicate that bodily location determines which bacteria are present as well. Naik’s 2020 study depicts a greater abundance of *Cutibacterium* in the inframammary and axilla regions in comparison with the inguinal and gluteal crease in healthy patients [10]. For HS patients who are naïve to treatment, an overall increase in anaerobic bacteria and a decrease in commensal bacteria can be observed [10,13,14].

Anaerobic bacteria have been known to cause skin abscesses in other contexts and have been isolated in several studies. A study conducted by Lapins et al. utilized CO_2_ laser ablation during surgery, which theoretically removed possible superficial commensal contaminants in the process. This led to the identification of anaerobic cocci in deep axillary and inguinal lesions [15]. Another study found *Fusobacterium* spp. and *Bacteroides* spp. to be associated with chronic lesions using metagenomics [16].

Historically, gram-positive cocci and gram-positive rods such as *Staphylococcus aureus*, CoNS, and *Corynebacterium* spp. have been considered to play major roles in HS. *S. aureus* specifically has been linked in studies to heavy smokers, and it is theorized that the use of nicotine may contribute to pathogen growth [17]. Metagenomic studies found increased anaerobes, increased *Corynebacterium*, and decreased *Cutibacterium* within active HS lesions, which aligns with past studies that used either deep or superficial cultures [18]. Estimations have shown that only 1% of bacteria are capable of being grown on cultures, thus making it difficult to detect the presence or absence of certain bacteria to accurately study the microbial role in HS pathogenesis. Culturing anaerobic bacteria is particularly difficult as it is easily harmed by oxygen [19]. Additionally, many species can only be grown on specific mediums, so cultures should mainly be used for confirmation or as supporting data. In 2014, Guet-Revillet et al. discovered a positive correlation between the abundance of anaerobic bacteria presence with Hurley staging and severity of disease. For Hurley stages II and III, the bacteria identified included strict anaerobes, *milleri* group *streptococci*, and anaerobic actinomycetes. More specifically, predominant gram-positive cocci identified were *Anaerococcus* spp., *Peptoniphilus* spp., and *Finegoldia* spp., and the predominant gram-negative rods identified were *Prevotella* spp., *Porphyromonas* spp., *Bacteroides* spp., and *Fusobacterium* spp. [20].

Despite these precedents, a study by Jahns et al. was not able to isolate *S. aureus* or CoNS in a retrospective study featuring 27 HS patients located in Sweden [21]. The study utilized syringes in order to collect skin samples from deeper cutaneous layers, thus avoiding bacterial contamination from superficial layers. This may be a reason *S. aureus* was not detected in this specific study. A study by Lapins et al., which also excluded superficial contamination, found less *S. aureus* in comparison to CoNS and overall that it had a decreased presence in deep lesions compared to the surface. This supports the theory that *S. aureus* is mainly found on the surface of the skin as a potential contaminant and is thus detected by swabs, which are a popular but limited superficial technique used in HS studies [15]. In addition, Guet-Revillet et al. detected *S. aureus* in 0% of Hurley stage I lesions, 4% of Hurley stage II lesions, and 25% of Hurley stage III lesions. This data support *S. aureus* as a bacteria that is mainly present in chronic, severe HS lesions [16,20]. Areas where the skin barrier is chronically compromised (nodules, cysts, and sinus tracts) may enable *S. aureus* to contaminate deeper into the skin as HS progresses over time.

CoNS is recognized as a skin commensal but is also known to cause severe infection in immunosuppressed patients. 16 out of 21 of the participants in the study by Lapins et al. had samples positive for CoNS, and 9 out of those 16 were positive only for CoNS [15,17]. A study by Sartorius et al. found similar results, with CoNS being the most predominant species in HS lesions. However, none were found in severe lesions using fluorescence in situ hybridization [16]. Although contamination is a possibility, further investigation needs to be conducted on the potential turning point of CoNS as a commensal bacteria into a pathogen.

*Staphylococcus lugdunensis*, a species of CoNS that mimics *S. aureus*, is known to induce abscesses and is mainly located on the breasts and buttocks. Recent studies suggested that *S. lugdunensis* is generally associated with abscesses in the pelvic region, with its preferred carriage site being the perineal and inguinal regions. The breast region may be an additional carriage site. In recent dermatological studies, *S. lugdunensis* was identified in 58% of HS nodules and abscesses [22]. Unlike other CoNS species, *S. lugdunensis* is established as having an association with Hurley stage I nodules and abscesses [16,20]. As mentioned, Guet-Revillet performed a microbiological analysis on HS patients and categorized them by Hurley stage. The results revealed all Hurley stage I patients had *S. lugdunensis* as the predominant culture while Hurley stages II and III reflected mixed anaerobe profiles [20]. It is possible that *S. lugdunensis* plays a role in the early disease process of HS.

### 2.2. Bacteria as a Secondary Event in HS Pathogenesis

There is still much ambiguity surrounding whether a paradigm shift in bacterial microflora is a primary or secondary event in HS. Clinicians routinely prescribe antibiotic therapy to their HS patients; however, it is not a cure and often only provides temporary suppression for these individuals [1,23]. The change in skin microbiome may not only be a cause but also an ongoing effect of underlying physiology. A localized immunodeficiency may enable commensal bacteria to turn on its host. Additionally, biofilm and bacterial colonization found in sinus tracts offer the potential for secondary infection [1].

### 2.3. The Role of Bacterial Biofilms in HS Disease

Several studies questioned whether or not HS should be redefined as a biofilm disease. In general, biofilms are associated with areas such as sinus tracts, which do not occur until later stages of HS pathogenesis. *S. epidermidis* in particular is known to form biofilm, especially in indwelling foreign devices such as catheters and prostheses [17]. Ring et al. investigated the microbiome of sinus tracts in moderate and severe HS patients and found that it was heavily dominated by anaerobic species such as *Prevotella* and *Porphyromonas* [18]. Antibiotics that target anaerobic bacteria include metronidazole, a nitroimidazole with broad-spectrum anaerobic activity coverage, and ertapenem, a carbapenem with resistance rates less than 1% [23]. CoNS has also been identified in biofilms found in severe HS patients [18].

In a case study by Kathju et al., a 47-year-old woman with a 20-year diagnosis of HS in her buttocks, perineum, and groin was found to have multiple abscesses and sinus tracts during surgery. Tissue samples were examined under confocal microscopy and revealed that bacteria were adherent to the sinus tissue and present in clusters at specific anatomic sites. In addition, the disease was unable to be eradicated by antibiotics. These characteristics align with Parsek’s and Singh’s criteria for biofilm disease; and thus HS may fall under this category [22,24].

As biofilm is mainly found in sinus tracts reported during later and more severe stages of HS, HS probably does not start as a biofilm disease but likely progresses into one. Moderate to severe HS, such as those categorized as Hurley stage II and III, appear to fit the criteria outlined by former precedents. Going forward, we should either consider viewing HS as a spectrum of disease or acknowledge a paradigm shift during the later stages of HS into a biofilm disease.

By nature, biofilms are microbial communities that protect the bacteria it harbors due to their strong tolerance to external stressors including antibiotic treatments. It enables these microbes to survive within the human body, leading to persistent inflammation and infection. There have been studies supporting a high level of metabolic activity on the outer portion of biofilm, and a lower level on the inner part. Antibiotic tolerance of some bacteria may be due to using antibiotics that target bacterial growth processes in areas of slow metabolism. In addition, it is also known that biofilms feature a gradient of oxygen within them that may be incompatible with certain antimicrobials. It is also a possibility that some antibiotics are not able to penetrate biofilms [25]. As a diagnosis that is often missed, it is important to further educate clinicians, especially those in primary care and emergency medicine settings, to recognize HS in its earlier stages prior to biofilm formation. Doing so may contribute not only to prevention of disease progression and biofilm formation, but may also serve as a prompt for patients to make crucial lifestyle changes such as smoking cessation and weight loss. Table 1 and Table 2 summarize the current understanding of microbial activity in HS.

### 2.4. Antibiotic Efficacy and Resistance in HS

Antibiotics are used by clinicians to treat HS due to their dual antibacterial and anti-inflammatory properties. In terms of treatment, topical clindamycin can be used for mild to moderate HS in Hurley stages I and II, but recurrent exacerbations can indicate a switch to a systemic antibiotic such as a tetracycline class antibiotic (e.g., doxycycline, minocycline, sarecycline) [1,28]. Second-line therapy for mild to moderate HS or first-line therapy for severe HS is a combination therapy of clindamycin and rifampicin (each 300 mg BID) [1]. The two work synergistically to increase bactericidal activity and to prevent potential resistance. Together, they cover a wide range of bacteria as clindamycin typically treats gram-positive and anaerobic organisms and inflammatory nodules, while rifampicin works to target both gram-positive and gram-negative bacteria as well as biofilm, abscesses, and granulomas [29].

van Straalen et al. organized a 12-week multicenter prospective study comparing the efficacy of tetracyclines (tetracycline, doxycycline, or minocycline) and clindamycin with rifampicin. First-generation (tetracycline) and second-generation (doxycycline, minocycline) tetracyclines are broad-spectrum antibiotics that cover a variety of gram-positive and -negative bacteria throughout the body, including aerobic bacteria and many atypical pathogens. Collectively, 40.1% of patients treated with an oral tetracycline and 48.2% of patients treated with a clindamycin and rifampicin combination achieved Hidradenitis Suppurativa Clinical Response (HiSCR) 50. By definition, this milestone represents at least a 50% reduction in total abscess and nodule count without an increase in abscess or draining fistula count relative to the patient’s baseline. Although efficacy was demonstrated for both treatments, the study may contain several confounding factors due to variability in validated outcomes [30].

Although it is considered to be a third-line option, dapsone is an antineutrophilic and antieosinophilic antibiotic that is also a possibility for those with mild-to-moderate HS without a G6PD deficiency [31,32]. A 2021 retrospective study features 56 patients with mostly mild to moderate disease and were prescribed 50–150 mg/day of dapsone. A total of 62.5% of patients achieved HiSCR50 after 12 weeks of treatment. Analysis from the study also identifies an association between tract presence and non-response risk [31]. These results support dapsone as a treatment for patients with mild to moderate HS that have not developed fistulas or sinus tracts.

Combination therapies are the gold standard when looking to combat antimicrobial resistance [1,28,29]. However, due to its high resistance rates, clindamycin needs reevaluation as a first-line therapy. In a study testing antibiotic resistance using 114 positive bacterial cultures from HS patients, Bettoli et al. found a prevalence of resistance of 65.7% for clindamycin and even 69.3% for rifampicin when not used synergistically with the former [26]. In a separate study by Hessam et al. testing antimicrobial resistance using cultures from deep HS lesions, the rate of resistance to clindamycin was about 55% [27]. This may exclude clindamycin and rifampicin combination therapy as an option for patients with progressive HS that develop such resistance. Additionally, biofilm formation has been known to serve as a virulence factor for certain bacteria. It is a possibility that in the context of HS, the presence of biofilm may contribute to antibiotic resistance [19]. If all antibiotic treatments have failed, biologics such as adalimumab, secukinumab, or infliximab can be considered [32]. Table 3 summarizes antibiotic monotherapy resistance rates determined by cultures from HS lesions.

### 2.5. Biologic Use and Innovation in HS

In the case that systemic antibiotics are not effective, clinicians may choose to recommend biologics for their patients either as a monotherapy or with concomitant antibiotic use. TNF-α has been identified in higher concentrations in HS skin and is thought to be a primary driver of the HS inflammatory process. For this reason, clinicians have turned to TNF-α inhibitors, more specifically adalimumab, which is currently one of two FDA-approved biologics for moderate to severe HS [32]. It works by selectively binding to TNF-α and inhibiting its interaction with cell surface receptors p55 and p75 [33].

The other biologic approved by the FDA for HS is the IL-17A inhibitor secukinumab. IL-17A has also been identified at high concentrations in HS skin and is thought to play a role in HS inflammation in addition to TNF-α. As an inhibitor, secukinumab binds selectively to IL-17A and thus neutralizes it [34]. At its baseline, IL-17 can protect the skin from bacterial pathogens such as *S. aureus*. Upon dysregulation or chronic activation, IL-17 has the potential to cause pathogenic inflammation [35]. It is also possible that *S. aureus* competes with CoNS, pathogens strongly represented in HS lesions and have also been shown to secrete antimicrobials that directly attack *S. aureus*. An IL-17 inhibitor may assist in resolving this microbial dysbiosis [36].

In Kimball’s PIONEER I and II trials, 307 patients and 326 patients were treated for HS with adalimumab, respectively. By week 12, PIONEER I boasted HiSCR50 rates of 41.8% for those patients receiving adalimumab versus 26.0% for those receiving a placebo. In PIONEER II, response rates were 58.9% for those patients receiving adalimumab versus 27.6% for those receiving placebo [37]. Overall, the study provided evidence of adalimumab’s efficacy against HS in comparison with placebo.

Additionally, Kimball’s SUNSHINE and SUNRISE clinical trials demonstrated positive benefits of IL-17A inhibitor secukinumab in treating moderate to severe HS when administered every 2 weeks. This study featured moderate to severe HS patients, with 541 participants and 543 participants, respectively. In the SUNSHINE trial, 45% of patients treated with secukinumab achieved HiSCR50 compared with 34% of the placebo group. 42% of patients treated with secukinumab achieved the same status compared with 31% treated with placebo in the SUNRISE trial [34].

Additional clinical studies showed positive responses from HS patients to the dual IL-17A and IL-17F inhibitor bimekizumab. Bimekizumab binds selectively to IL-17A, IL-17F, and the IL-17A/F heterodimer, ultimately inhibiting the associated inflammatory cascade [38]. Glatt’s phase II clinical trial featured 73 participants with moderate to severe HS who were treated with either bimekizumab, placebo, or adalimumab as the reference arm. At week 12, the HiSCR50 rate was higher for the group treated with bimekizumab at 57.3% in comparison with the placebo group who achieved an HiSCR50 rate of only 26.1% [39]. As of 2023, bimekizumab entered phase III clinical trials BE HEARD I and BE HEARD II featuring 505 patients and 509 patients, respectively. These studies evaluated bimekizumab 320 mg every 2 weeks (Q2W) and bimekizumab 320 mg every 4 weeks (Q4W) against placebo. At 16 weeks, 47.8% of patients treated with bimekizumab Q2W, 45.3% of patients treated with bimekizumab Q4W, and 28.7% of patients treated with a placebo achieved HiSCR50 in BE HEARD I. In BE HEARD II, the same treatment groups of patients achieved rates of 52%, 53.8%, and 32.2%, respectively. Overall, over 55% of patients treated with bimekizumab achieved a HiSCR75 by week 48 (75% reduction in abscess and nodule count) [40].

### 2.6. Janus Kinase Inhibitors for the Treatment of HS

Although it is not currently part of the standard of care, there is emerging research in relation to Janus kinase (JAK) inhibitors and their treatment of HS. JAK inhibitors work by reducing inflammation and controlling disease severity and have been approved for use in other dermatological disorders including alopecia areata, atopic dermatitis, vitiligo, and psoriasis [41]. There also appears to be a relationship between multiple JAK-mediated cytokines that could potentially be targeted [41].

Alavi et al. tested the efficacy of JAK1 inhibitor povorcitinib with two separate phase II studies. Study 1 was a single-arm study treating seven patients with moderate to severe HS with 15 mg daily for 8 weeks. Study 2 consisted of 33 patients in randomized cohorts treated with 30 mg, 60 mg, 90 mg, and a placebo for 8 weeks. A total of 43% of patients from study 1 achieved HiSCR50 at week 8. In study 2, 56% of patients taking 30 mg, 56% taking 60 mg, 88% taking 90 mg, and 57% receiving placebo were able to achieve a HiSCR50 at week 8 [41]. There appears to be proof of efficacy for povorcitinib as a treatment for HS, although its evaluation needs to be repeated with larger cohort sizes.

In a study analyzing punch biopsies from HS patients treated with povorcitinib, results showed upregulated genes involving the sweat gland compared to their downregulation before treatment. These genes included WIF1, KRT77, and KRT31, which are involved in sweat gland development, as well as FOXA1, which is involved in sweat secretion and is upregulated in wound-healing skin [42]. Increased perspiration has been reported by HS patients, which could be an effect of sweat gland dysfunction and potentially contribute to sweat retention. This may be related to follicular plugging, thus leading to the accumulation of cytokines and bacteria [43]. Povorcitinib is currently undergoing phase III clinical trials for HS [44].

Kozera et al. also conducted a retrospective study of JAK1-selective inhibitor upadacitinib, which was used in 20 patients with moderate to severe HS. Participants were treated with 15 mg per day, and those that did not achieve HiSCR50 at week 4 were increased to a 30 mg dose. Overall, 75% achieved HiSCR50, 30% achieved HiSCR75, and 20% achieved HiSCR90 at week 4 (90% reduction in abscess and nodule count). At week 12, 100% achieved HiSCR50, 95% achieved HiSCR75, and 30% achieved HiSCR90. However, 20% of participants reported new abscesses that were more severe in comparison to previous flares [45]. Although there appears to be success with the utilization of upadacitinib in patients with HS, researchers and clinicians should consider a randomized, double-blind, placebo-controlled trial with a larger cohort to address possible confounding factors. As of 2023, upadacitinib is currently undergoing phase III clinical trials for HS [46].

### 2.7. IRAK4 Degraders in HS

In a different study analyzing biopsies of HS lesions with quantitative real-time PCR, IL-1β was found to be extremely elevated and IL-1α to be mildly elevated as well. It is hypothesized that the high amount of IL-1β (and possibly even IL-1α) could trigger inflammatory cascades that could lead to purulent discharge, a key symptom of HS, as well as follicle plugging [47].

IL-1 receptor-associated kinase 4 (IRAK4) is a protein kinase essential for signaling pathways that lead to the introduction of proinflammatory cytokines found in HS [47]. Recent data from a Phase I trial by Kymera Therapeutics support IRAK4 degrader KT-474 as a potential HS treatment due to its results and impact on inflammation. Out of 12 HS patients, 10 with moderate to severe disease and 2 with severe disease, 42–50% achieved HiSCR50. Results were observed across 28 days followed by a 2-week follow-up [48]. KT-474 is currently in phase II for its treatment of HS [49].

### 2.8. Novel Biologic Interleukin Antagonists for HS

Lutikizumab is a dual-variable-domain interleukin 1α/1β antagonist that inhibits IL- 1α and IL-1β, which are two prominent proinflammatory cytokines in HS. Results were recently released for a phase II trial featuring 153 HS patients, with the majority having Hurley stage 3 patients. These patients were treated with 100 mg Lutikizumab, 100 mg every other week, 300 mg every week, 300 mg every other week, or a placebo. In terms of achieving HiSCR50 and HiSCR75, this study demonstrated higher response rates for patients treated with Lutikizumab 300 mg weekly (Q1W) and every other week (Q2W) compared to the other groups. At week 16, the HiSCR50 response rates for patients treated with 100 mg Q2W, 300 mg Q1W, 300 mg Q2W, and a placebo were 27%, 48.7%, 59.5%, and 35%, respectively. Within the same timeframe, 16.2%, 38.5%, 45.9%, and 17.5% of those same groups were able to achieve HiSCR75 [50]. Table 4 compares HS therapies and their respective HiSCR50 (and higher in some cases) achievements.

### 2.9. Future Directions in Understanding Bacteria in HS Pathogenesis and Treatment

In order to further our medical knowledge of HS as it relates to the skin microbiome, studies must be conducted with standardized methodologies, such as stratifying skin samples into their respective layers and utilizing syringes and CO_2_ laser ablation during sample collections to avoid superficial bacterial contamination. To maintain consistency, samples from different disease sites such as the axillary, genital, and perineal areas should only be compared to themselves due to differences in the topographical microbiome. There is also a disparity concerning the different microbiomes that are reflected when using swabs, biopsies, and scrapes [17]. Sample collections should therefore be standardized in order to obtain consistent and comparable data.

Although bacterial cultures have been predominantly used in the past, future cultures should mainly be used as supporting data for other types of advanced sampling technologies as the majority of bacteria are unable to be cultured due to specific growth requirements including the absence of oxygen. If utilized in future studies, then they should be incubated for longer periods of time, either with or without oxygen depending on individual bacteria requirements, to allow certain species of bacteria to grow effectively. Furthermore, newer and more informative technologies such as metagenomics and RNA gene sequencing should be the main methodology when it comes to identifying present bacteria.

For HS patients displaying higher rates of bacterial infections requiring antibiotics, more study is needed to understand the baseline antibiotic resistance rate among this HS patient population and its correlation with bona fide tissue infection. Immunomodulators, antibiotics, and biologics do not treat dysbiosis. Conversely, they further amplify it and consequently do not always alter the patient’s microbiological or overall HS journey. New therapies aimed at curing HS should also target dysbiosis (i.e., probiotics, postbiotics, and prebiotics) to decrease flares and prevent tissue inflammation.

## 3. Conclusions

HS overall remains an ambiguous and heterogeneous disease that requires more research in order to solidify current theories of its bacterial and inflammatory pathogenesis and to discover new schools of thought. One area of needed emphasis is better understanding the role of pilosebaceous unit anaerobes in driving tissue inflammation and destruction. For HS in Hurley stages II and III, clinicians may consider shifting their focus mainly to anaerobes. As the rate of resistance grows for clindamycin and other antibiotics like the tetracycline class, it may be appropriate and necessary to use antibiotic combinations in HS treatments to reduce the risk of antibiotic resistance.

As there is ample room for therapeutic innovation in HS, it is also possible, if not likely, that HS treatment will pivot away from antibiotics for moderate to severe disease and become reliant on biologics and/or advanced systemic small-molecule therapies as more conclusive clinical studies are published. Newer and/or re-purposed biologics and small-molecule therapies offer encouraging opportunities for improving both HS patient outcomes and promoting antibiotic stewardship. For a heterogenous disease like HS, with a suspected multi-factorial pathogenesis, it is likely that in the future, a disease-modifying or remittive therapy will combine an advanced/targeted anti-inflammatory approach with one that effectively modulates cutaneous and deep tissue dysbiosis.

## Figures and Tables

**Table 1:** HS-specific skin findings featured in this review.

Study	HS Findings
Naik et al. (2020) [10]	Higher number of mixed anaerobes and diversity; possible correlation between presence of anaerobes and disease severity; decreased *Cutibacterium* overall but greater presence in inframammary and axilla compared to healthy patients
Wolk et al. (2020) [14]	Presence of anaerobic cocci in deep axillary and inguinal lesions; less *S. aureus* in comparison to CoNS; decreased *S. aureus* presence in deep lesions compared to the surface
Nikolakis et al. (2015) [16]	*Fusobacterium* spp. and *Bacteroides* spp. may be associated with chronic lesions; did not detect CoNS in severe lesions
Ring et al. (2015) [17]	Most predominant species detected in HS lesions appears to be CoNS, and in some cases, was the only detected bacteria; sinus tracts are heavily dominated by anaerobic species (ex. *Prevotella*, *Porphyromonas*)
Williams et al. (2020) [18]	Increased anaerobes and *Corynebacterium*, and decreased *Cutibacterium* within active lesions; CoNS identified in biofilm of severe HS patients
Guet-Revillet et al. (2014) [20]	Possible positive correlation between abundance of anaerobic bacteria and severity of disease; identified strict anaerobes, *milleri* group *streptococci*, and anaerobic actinomycetes; predominant gram-positive cocci were *Anaerococcus* spp., *Peptoniphilus* spp., and *Fineholdia* spp. and predominant gram-negative rods were *Prevotella* spp., *Porphyromonas* spp., *Bacteroides* spp., and *Fusobacterium* spp.; *S. aureus* detected in 0% of Hurley stage I lesions, 4% of stage II lesions, and 25% of stage III lesions; *S. lugdunensis* is predominant in Hurley stage I, and stages II and III have mixed anaerobe profiles
Jahns et al. (2014) [21]	Unable to isolate *S. aureus* or CoNS from deeper cutaneous layers
Kathju et al. (2012) [23]	Featured case study showcases chronic and severe HS with developed biofilm that is unable to be eradicated by antibiotics
Bettoli et al. (2019) [26]	Evaluated antibiotic resistance rates were 65.7% for clindamycin and 69.3% for rifampicin when not used synergistically
Hessam et al. (2016) [27]	Evaluated antibiotic resistance rate for clindamycin was about 55%

**Table 2:** Established bacteria in HS and their known roles and relationships.

Study	Bacteria	Role	HS Relationship
Naik et al. (2020) [10], Shu et al. (2013) [11]	*Cutibacterium*	Skin commensal that ferments carbohydrates into propionic acid, which is a compound known for its bactericidal activity against pathogens; metabolizes free fatty acids from sebum and decreases surface pH to support commensal bacteria while inhibiting pathogens and preventing biofilm formation	Decreased overall but greater presence in inframammary and axilla compared to healthy patients
Wolk et al. (2020) [14], Nikolakis et al. (2015) [16], Ring et al. (2015) [17]	Coagulase-negative *Staphylococci*	Skin commensal that can also cause severe infection in immunosuppressed patients	The most predominant species detected in HS lesions according to some studies; none detected in severe lesions when fluorescence in situ hybridization is utilized
Nikolakis et al. (2015) [16], Guet-Revillet et al. (2014) [20], Molinelli et al. (2023) [22]	*Staphylococcus lugdunensis*	Species of CoNS that mimics *S. aureus* and is known to induce abscesses; preferred carriage site appears to be the perineal and inguinal regions with the breast region as an additional site	Detected in HS nodules and abscesses; established association with Hurley stage I
Ring et al. (2015) [17]	*Staphylococcus epidermidis*	Known to form biofilms, especially in indwelling foreign devices	Present in sinus tracts

**Table 3:** Monotherapy resistance rates of organisms collectively found in HS lesions to HS treatment-related antibiotics.

Study	Drug	Methodology	Resistance Rate	Most Commonly Isolated Bacteria
Bettoli et al. (2019) [26]	Clindamycin Rifampicin Penicillin Ciprofloxacin Tetracycline Erythromycin	Cultured purulent material from HS lesions collected from swabs	65.7% 69.3% 70.0% 74.0% 84.7% 89.0%	Bacterial families: Enterobacteriaceae (30.7%), *Staphylococcus* (25.2%), *Streptococcus* (14.1%) Genus or species: *Proteus* spp. (13.5%), *Escherichia coli* (9.8%)
Hessam et al. (2016) [27]	Clindamycin	Cultures from deep portions of HS lesions obtained from surgical patients	55.0%	Coagulase-negative *Staphylococci*, *Staphylococcus aureus*, *Proteus mirabilis*, *Escherichia coli*

**Table 4:** Antibiotics, biologics, JAK Inhibitors, and IRAK4 degraders used in HS and the HiSCR rates achieved in clinical trials to date.

Study	Drug	Achieved HiSCR50 Rate
van Straalen et al. (2021) [30]	Oral Tetracyclines Clindamycin with Rifampicin	Week 12: 40.1% Week 12: 48.2%
López-Llunell et al. (2021) [31]	Dapsone	Week 12: 62.5%
Kimball et al. (2016) [37]	Adalimumab	PIONEER I: 41.8% Adalimumab vs. 26.0% placebo PIONEER II: 58.9% Adalimumab vs. 27.6% placebo
Kimball et al. (2023) [34]	Secukinumab	SUNSHINE: 45% Secukinumab vs. 34.0% placebo SUNRISE: 42% Secukinumab vs. 31.0% placebo
Glatt et al. (2021) [39]	Bimekizumab	57.3% Bimekizumab vs. 26.1% placebo
Kimball et al. (2023) [40]	Bimekizumab	BE HEARD I Week 16: 47.8% Bimekizumab Q2W vs. 45.3% Bimekizumab Q24 vs. 28.7% placebo BE HEARD II Week 16: 52% Bimekizumab Q2W vs. 53.8% Bimekizumab Q24 vs. 32.2% placebo (BE HEARD I/II Week 48 HiSCR75: 55.0% Bimekizumab)
Alavi et al. (2022) [41]	Povorcitinib	Study 1: 43.0% Povorcitinib Study 2: Total 65.0% Povorcitinib (56.0% 30 mg, 56.0% 60 mg, 88.0% 90 mg) vs. 57.0% placebo
Kozera et al. (2022) [45]	Upadacitinib	Week 4: 75.0% Upadacitinib 15 mg (30.0% HiSCR75, 20.0% HiSCR90) Week 12: 100% Upadacitinib 30 mg (95.0% HiSCR75, 30.0% HiSCR90)
Ackerman et al. (2023) [48]	KT-474	28 days with 2-week follow-up: 42.0–50.0%
PR Newswire (2024) [50]	Lutikizumab	Week 16: 27.0% Lutikizumab 100 mg Q2W vs. 48.7% Lutikizumab 300 mg Q1W vs. 59.5% Lutikizumab 300 mg Q2W vs. 35.0% placebo (Week 16 HiSCR75: 16.2% Lutikizumab 100 mg Q2W vs. 38.5% Lutikizumab 300 mg Q1W vs. 45.9% Lutikizumab 300 mg Q2W vs. 17.5% placebo)

## Data Availability

All data is available within cited primary articles.
